# Midterm outcomes of early versus late surgery for infective endocarditis with neurologic complications: a meta-analysis

**DOI:** 10.1186/s13019-021-01425-x

**Published:** 2021-03-25

**Authors:** Yujiro Yokoyama, Taichiro Goto

**Affiliations:** 1 Department of Surgery, St. Luke’s University Health Network, Bethlehem, PA USA; 2 Department of General Thoracic Surgery, Yamanashi Central Hospital, Yamanashi, 400-8506 Japan

**Keywords:** Infective endocarditis, Early surgery, Neurologic complication, Stroke, Cerebral infarction

## Abstract

**Background:**

Cerebral infarction (CI) remains one of the most common and fatal complications of infective endocarditis (IE), and the timing of surgery for IE with neurologic complications is controversial. As outcomes beyond the perioperative period have not been assessed with a meta-analysis previously, we conducted a meta-analysis comparing mid- to long-term outcomes of early and late surgery in patients with IE and neurologic complications.

**Methods:**

All studies that investigated early and late surgery in patients with IE and neurologic complications were identified. The primary and secondary endpoints were all-cause mortality and recurrence, respectively. Hazard ratios (HRs) for all-cause mortality and recurrence were extracted from each study.

**Results:**

Our search identified five eligible studies, which were all observational studies consisting of a total of 624 patients with IE and neurologic complications. Pooled analyses demonstrated that all-cause mortality was similar between the early and late surgery groups (HR [95% confidence interval [CI]] = 0.90 [0.49–1.64]; *P* = 0.10; I^2^ = 49%). Similarly, the recurrence rates were similar between both groups (HR [95% CI] = 1.86 [0.76–4.52]; *P* = 0.43; I^2^ = 0%).

**Conclusions:**

Our meta-analysis showed similar mortality and recurrent rates between the early and late surgery groups. The optimal timing of surgery should be individualized on a case-to-case basis.

## Background

Cerebral infarction (CI) is still one of the most common and fatal complications of infective endocarditis (IE). The incidence of CI varies from 10 to 40% [1, 2], with an increased risk of mortality [3, 4]. The timing of surgery for IE with neurologic complications remains controversial since early surgery might result in conversion into hemorrhagic stroke as a result of systemic anticoagulation during cardiopulmonary bypass, or the exacerbation of an ischemic stroke from hypotension during surgery, although late surgery might result in continued neurological insult from embolic events. A previous meta-analysis showed that early surgery was associated with a significant increase in perioperative mortality and neurological deterioration in patients with IE complicated with neurologic events [5]. However, the outcomes beyond the perioperative period were not analyzed as many studies did not investigate long-term outcomes or just provided Kaplan-Meier curves without hazard ratios [HRs]. We previously reported a method to extract HRs from Kaplan-Meier curves [6]. By using this method and updating the available studies, we conducted a meta-analysis comparing mid- to long-term outcomes of early and late surgery in patients with IE complicated with neurologic events.

## Methods

All studies that investigated early and late surgery in patients with IE and neurologic complications were identified using a two-level strategy. First, databases including MEDLINE and EMBASE were searched on July 27, 2020, using Web-based search engines (PubMed and OVID). Second, relevant studies were identified through a manual search of secondary sources, including references of initially identified articles, reviews, and commentaries. All references were downloaded for consolidation, elimination of duplicates, and further analyses. Search terms included “endocarditis,” “early or timing,” and “surgery or surgical.” We did not apply language limitations. Two independent and blinded authors (Y.Y. and T.G.) reviewed the search results separately to select the studies based on present inclusion and exclusion criteria. Disagreements were resolved by means of a consensus-based discussion.

The included studies met the following criteria: the study was a randomized controlled trial or an observational study, the study population consisted of patients with IE and neurologic complications including CI or intracranial hemorrhage, the enrolled patients were assigned to the early surgery group and the late surgery group, and the outcomes included all-cause mortality with more than 6 months of follow-up. The definitions of early and late surgery were according to each study.

The primary endpoint was all-cause mortality, and the secondary endpoint was recurrence. The review was conducted according to the Preferred Reporting Items for Systematic Reviews and Meta-Analyses statement standards [7]. HRs for all-cause mortality and recurrence were extracted from each study. The adjusted HR was extracted if available. If the HR was not described in a study, then the HR was calculated from the Kaplan-Meier curve if available using the “HR-calculation spreadsheet” developed by Tierney et al. [8] based on standard statistical methods reported by Palmar et al. [9] and Williamson et al. [10]. If a Kaplan-Meier curve was not provided in a study, then the risk ratio was calculated from the event number and the patient number. Review Manager (RevMan) Version 5.3 (Nordic Cochrane Centre, the Cochrane Collaboration, 2012, Copenhagen, Denmark) was used to combine HRs in the random-effects model. The random-effects model was used in each outcome regardless of the heterogeneity among studies as it allowed for a more conservative assessment of the pooled effect size. ProMeta 3 software (https://idostatistics.com/prometa3/) was used to perform sensitivity analyses and examine funnel-plot asymmetry. Funnel-plot asymmetry suggesting publication bias was assessed mathematically using Egger’s linear-regression test [11]. Significant heterogeneity was considered to be present when the I^2^ index was over 50% or the P for heterogeneity was <.05. Sensitivity analyses were performed by eliminating one study at a time to confirm that our findings were not derived from any single study [12].

## Results

**Fig. 1:**
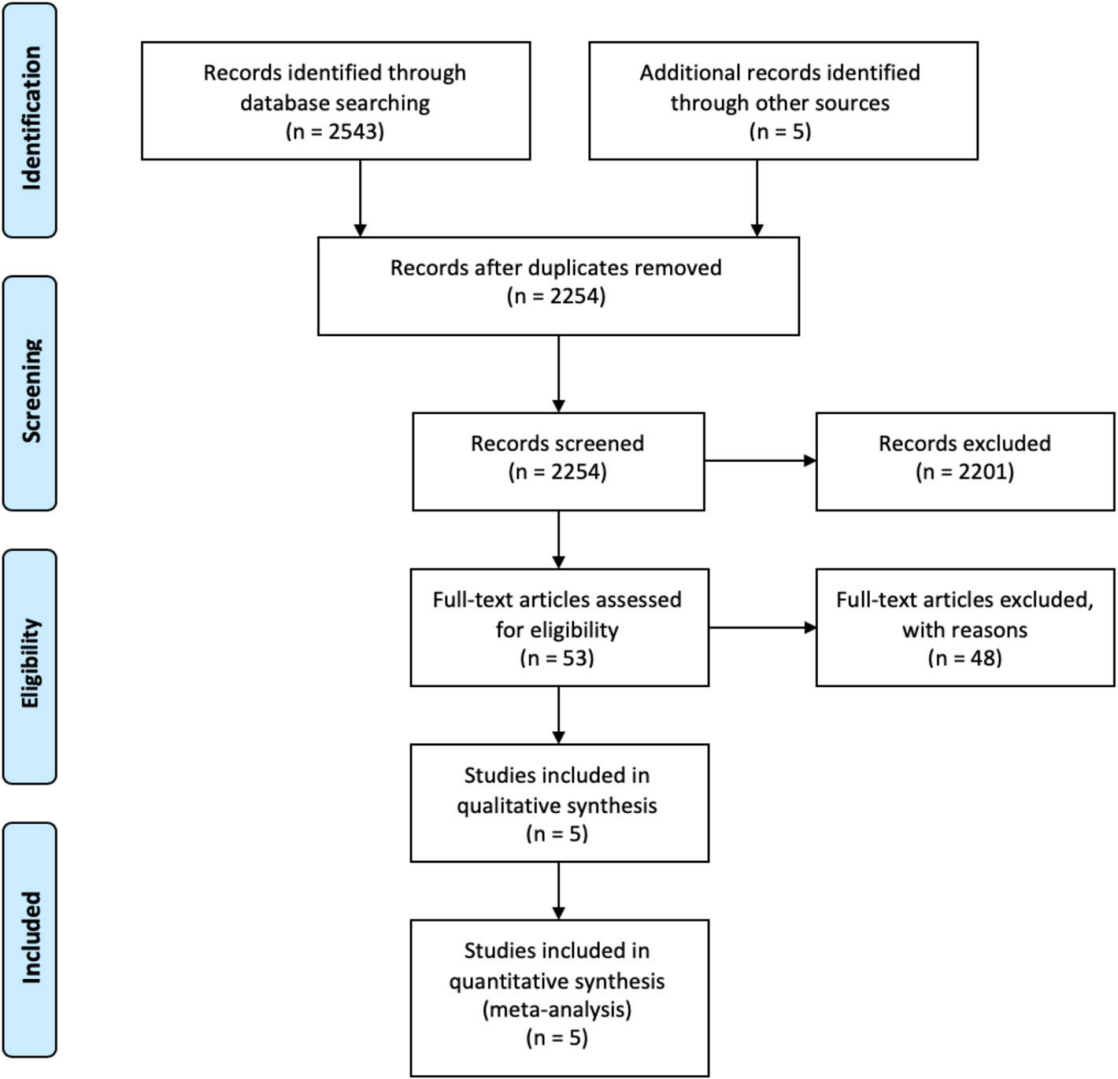
Workflow for the selection of eligible original papers according to the PRISMA (Preferred Reporting Items for Systematic Reviews and Meta-Analyses) criteria for our original meta-analysis

Our search identified five eligible studies [13–17], which were all observational studies consisting of a total of 624 patients with IE complicated with neurologic events assigned to the early surgery (*n* = 252) or late surgery groups (*n* = 372) (Fig. 1). The study profile and patient characteristics are summarized in Table 1.

**Table 1:** Summary of included studies

Author	Year	Follow-up (months)	Definition of early surgery	Mean or median time to surgery (day)		Patients (n)	Early (n)	Late (n)
			Early	Early	Late			
Thuny [13]	2009	6	< 7 days	3	19	241	95	146
Kim [14]	2011	62	< 14 days	3	20	56	34	22
Barsic [15]	2012	12	< 7 days	NA	NA	56	34	22
Oh [16]	2016	51	< 7 days	4	17	39	20	19
Samura [17]	2019	60	< 3 days	1	16	90	45	45
Age, median or mean ± SD		Female (%)		Diabetes (%)		CHF (%)		
Early	Late	Early	Late	Early	Late	Early	Late	
53 ± 16	58 ± 15	27	21	10	15	46	40	
44 ± 14	40 ± 16	32	36	9	14	NA	NA	
53	57	31	32	24	17	NA	NA	
53 ± 15	45 ± 15	25	42	10	5	50	42	
64	65	44	36	24	36	NA	NA	
Valve involved				Prosthetic valve (%)		Cerebral hemorrhage (%)		
Early		Late		Early	Late	Early	Late	
A-64%, M-53%, B-19%		M-70%, B-50%, T-22%		21	31	2	5	
A-35%, M-53%, B-12%		A-27%, M-55%, B-18%		3	27	14	41	
A-60%, M-43%		A-48%, M-58%		22	23	NA	NA	
A-55%, M-55%, T-5%		A-47%, M-79%		45	37	20	32	
A-45%, M-71%		A-45%, M-64%		19	21	0	0	

*A* Aortic valve, *B* Aortic and mitral valves, *CHF* Congestive heart failure, *M* Mitral valve, *NA* Not available, *T* Tricuspid valve

**Fig. 2:**
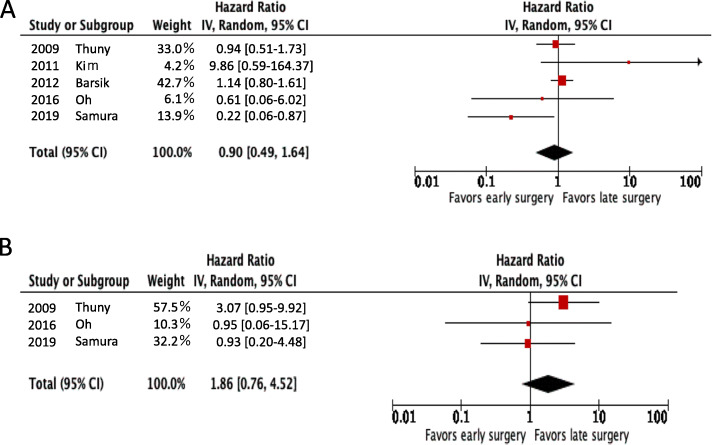
Comparison of all-cause mortality (**a**) and recurrence (**b**) between the early surgery and late surgery groups using the random-effects model in the original studies from the reviewed meta-analysis. The left portion of the figure shows the studies analyzed with their corresponding hazard ratios and lower and upper limits. The right portion of the figure shows a forest plot of the data. The horizontal lines represent the values within the 95% CI of the underlying effects. The vertical line indicates a hazard ratio of 1. CI = confidence interval; HR = hazard ratio; IV = inverse variance

Pooled analyses demonstrated that all-cause mortality was similar between the early and late surgery groups (HR [95% confidence interval [CI]] = 0.90 [0.49–1.64]; *P* = 0.10; I^2^ = 49%) (Fig. 2a). Likewise, the recurrence rates were similar in both groups (HR [95% CI] = 1.86 [0.76–4.52]; *P* = 0.43; I^2^ = 0%) (Fig. 2b).

**Fig. 3:**
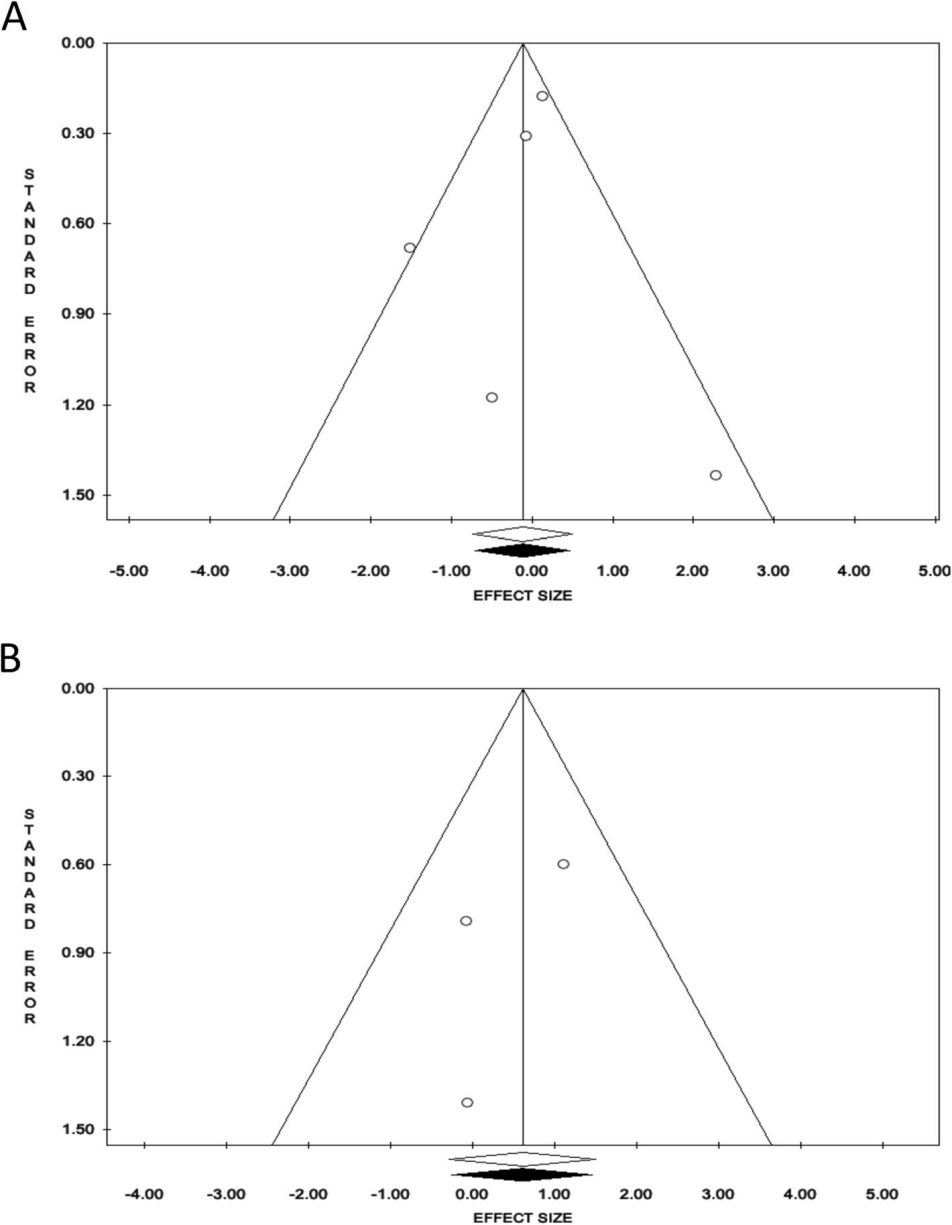
Funnel plot for each phase. **a** All-cause mortality, **b** recurrence

**Fig. 4:**
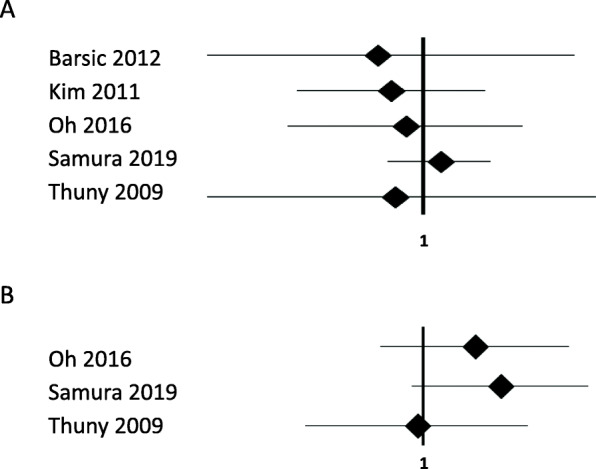
Sensitivity analyses eliminating one study at a time. **a** All-cause mortality; **b** recurrence

Publication bias was assessed using funnel plots (Fig. 3), which showed no evidence of publication bias in the analyses of all-cause mortality and recurrence, respectively (*P* = 0.75, 0.51). In terms of sensitivity analyses (Fig. 4), eliminating any single study did not change the primary results (pooling all the studies) of both all-cause mortality and recurrence.

## Discussion

This meta-analysis demonstrated that early and late surgery in patients with IE complicated with CI were associated with similar rates of all-cause mortality and recurrence. IE remains a major illness with a high mortality secondary to complications including congestive heart failure and neurologic events [18, 19]. Current guidelines recommend early surgery when specific parameters are met, such as recurrent emboli or large vegetation [20, 21]. A previous meta-analysis demonstrated that early surgery was associated with significant reduction in all-cause mortality in patients with IE [22]. However, early surgery might have an opposite effect after IE is complicated with neurologic events, since anticoagulation during cardiopulmonary bypass can turn an ischemic stroke into a hemorrhagic stroke, or exacerbate a stroke due to hypotension during operation. Current guidelines recommend delaying surgery for 3 to 4 weeks in IE patients with major or hemorrhagic stroke [23, 24]. Similarly, a recent meta-analysis showed that early surgery was associated with increased mortality and neurological exacerbation in patients with hemorrhagic stroke as well as ischemic stroke [5]. However, they were unable to analyze the outcomes beyond the perioperative period owing to scarcity of data on mid- to long-term outcomes.

This is the first meta-analysis comparing the outcomes beyond the perioperative period between early and late surgery in patients with IE complicated with CI. Although previous meta-analyses demonstrated worse perioperative mortality with early surgery than that with late surgery [5, 22], our results showed similar mortality and recurrence rates. Furthermore, leave-one-out sensitivity analysis showed that eliminating any one of those studies did not change the outcomes, suggesting that our findings were not derived from any single study. This could be attributed to the fact that 40–60% of patients with IE develop heart failure because of structural damage [25, 26], and early surgery might prevent the development of heart failure caused by progressive regurgitation [27]. Therefore, although early surgery might increase preoperative mortality and neurologic complications in patients with IE complicated with neurologic events [5], it might have a positive effect on long-term outcomes by preserving cardiac function.

Our analysis has several limitations. First, our study comprised observational studies and is therefore subject to possible selection bias. Second, we did not analyze perioperative outcomes, since a previous meta-analysis including 27 observational studies [5] was conducted and the perioperative outcomes were thoroughly analyzed. Although they could not analyze the outcomes beyond the perioperative period, we were able to analyze them by extracting the HR from the Kaplan-Meier curve [6]. Third, the definition of early surgery varied among the studies, ranging from 3 to 14 days. Finally, there might be survivor bias in the late surgery group, and we could not assess how many patients died while waiting for the surgery.

## Conclusions

Our meta-analysis showed similar mortality and recurrent rates between early and late surgery in patients with IE complicated with neurologic events. The optimal timing of surgery should be individualized on a case-to-case basis. Further trials with long-term outcomes are warranted.

## Data Availability

Not applicable.

## Abbreviations

CI: Cerebral infarction
IE: Infective endocarditis
HR: Hazard ratio
